# Effectiveness of health promotion intervention on the knowledge and selected practices related with oral cancer among a group of vulnerable youth in Sri Lanka

**DOI:** 10.1186/s12889-023-16298-z

**Published:** 2023-07-14

**Authors:** Manori Dhanapriyanka, Kanthi RDFC, Prasanna Jayasekara

**Affiliations:** 1 School of Dentistry, Faculty of Health and Behavioural Sciences, University of Queensland, Brisbane, Australia; 2 Ministry of Health Sri Lanka, No. 215 U/7, Anderson Flats, Park road, Colombo 05, Sri Lanka

**Keywords:** Intervention, Tobacco chewing, Youth, Oral cancer

## Abstract

**Background:**

There has been a noticeable trend of younger people being diagnosed with oral cancer, particularly among those from low socio-economic backgrounds. Poor knowledge on risk factors toward oral cancer and the growing fashion of using tobacco also identified among younger generation. Present study aimed to assess the effectiveness of a health promotion intervention to improve the knowledge and encourage positive practices associated with oral cancer among a group of vulnerable youth in Sri Lanka.

**Methods:**

The study was a community based quasi experimental study, conducted in urban slums in the district of Colombo, Sri Lanka. Sample size for one group was 120 youth participants aged between 15 and 24 years. Health promotion intervention was implemented to the intervention group and follow up period was 6 months. The control group did not receive the intervention. Awareness of oral cancer and oral potentially malignant disorders, tobacco chewing practice (betel quid chewing and commercially prepared tobacco and areca nut packet (CPTAP) chewing) and self-mouth examination practice were assessed at the beginning and after 6 months in both groups using an interviewer administered questionnaire. Changes in the knowledge, self-mouth examination practice, quit rate and fresh up take rate were computed to determine the effectiveness.

**Results:**

There was no loss to follow up. No significant difference was observed between the groups in pre intervention assessment regarding the knowledge, tobacco chewing and self-mouth examination practices. Knowledge score was significantly differed between the groups P = 0.000 in the post intervention assessment as well as among females P = 0.001. Quit rate of the tobacco chewing practice, betel chewing practice and CPTAP chewing practice among intervention group was 33%, 70%, and 13% respectively while control group did not have any quitters, P = 0.001. Fresh up take rate of tobacco chewing in the intervention group was 6.7% compared to the 37.5% in the control group, P = 0.001. Practicing self-mouth examination was significantly higher in intervention group in post intervention assessment, P = 0.000.

**Conclusion:**

Multicomponent health promotion intervention (Advocacy, Interactive discussions, IEC materials and Community mobilization) was significantly effective in enhancing the knowledge, increasing self-mouth examination practice, and reducing tobacco chewing practice among a vulnerable group of youth in Sri Lanka.

**Supplementary Information:**

The online version contains supplementary material available at 10.1186/s12889-023-16298-z.

## Introduction

Cancer is a leading cause of death, ranking as the second most common cause of death in developed countries and the third most common cause in developing countries. Globally, oral cancers accounts for 3% of all cancer cases [1]. According to the World Health Organization (WHO), in 2020 there were an estimated 377,713 new cases of oral cancer and approximately 177,756 deaths, placing it as the 13th most prevalent cancer globally. More than 50% of oral cancer cases occur in Asia, with around 11% of them originating from Southeast Asia [2, 3]. Oral cancer is the commonest cancer among males in Sri Lanka [4]. Further, there has been a noticeable trend of younger people being diagnosed with oral cancer, particularly among those from low socio-economic backgrounds [3]. This could be due to the growing fashion of using tobacco among younger generation [5–7]. While e cigarettes are becoming more common among youth in the western world [8], chewing tobacco is becoming commoner among the Southeast Asian region [9]. In India out of 184 million tobacco users, 40% used Smoke Less Tobacco (SLT) products [10]. According to the latest WHO non communicable disease risk factor survey conducted in Sri Lanka, there were 15.8% current SLT users [11]. Global Youth Tobacco Survey conducted in Sri Lanka, has identified 2.5% current SLT users in the year 2015 [12].

Moreover, oral cancers are often diagnosed at late stages. Delayed diagnosis of oral cancer can indeed have significant negative consequences such as complicated treatments, increase cost, lower survival rates, and reduced quality of life [13]. Majority of oral cancers develop from oral potentially malignant disorders (OPMD), which are identifiable stages characterized by visible changes in the oral cavity [1, 14]. Since the oral cavity is easily accessible and visible, individuals with knowledge and awareness can potentially identify early lesions and symptoms, such as changes in the oral cavity, red or white patches, and difficulty in opening the mouth [3]. Early identification of these signs allows for prompt treatment and further evaluation, which can significantly improve treatment outcomes [13]. Hence, increase awareness on oral cancer, oral potentially malignant disorders, and the importance of regular self-oral examinations are crucial in promoting early detection and reduction of tobacco use.

There are various interventions carried out to reduce the tobacco use globally. Successful community-based, peer-led, multicomponent tobacco intervention programmes were undertaken with adolescents and young adults in developed countries, but less were done among developing countries [15]. Kyle has identified in a pilot study, that one-hour long cancer specific educational interventions named “Let’s talk about it” was effective in raising adolescent’s awareness of cancer risk behaviours [16]. This study recommends that such kind of educational programmes must be conducted on a regular basis. A systematic review conducted on smokeless tobacco cessation interventions has identified that even though there are limited evidence on SLT cessation interventions globally, behavioural interventions are more suitable for low resource high SLT burden countries [17].

There are several intervention programmes conducted in Sri Lanka targeting to prevent tobacco behaviours as policy level interventions and health promotion interventions. In the past few years, the government has taken several giant leaps to restrict the use of tobacco by implementing a strong national-level action to enforce legislative measures against tobacco use including smokeless tobacco. Despite these, according to the latest National Cancer Control programme data in Sri Lanka revealed that oral cancer remains as a major public health concern making tobacco chewing as a foremost public health issue within the country [4]. A study conducted among youth living in urban slums in Sri Lanka uncovered concerning findings regarding tobacco use. The study revealed that 44.9% of the youth surveyed were current smokeless tobacco chewers, indicating a high prevalence of tobacco use among this population [18]. Furthermore, around 72% of the youth residing in urban slum areas in Sri Lanka, had poor knowledge related to oral potentially malignant disorders. As well as the study reported that only 1.2% of the youth surveyed had knowledge about self-mouth examination for oral cancer [19].

These findings underscore the importance of targeted interventions, public health initiatives, and comprehensive education programs to address tobacco use, improve knowledge about oral potentially malignant disorders, and promote self-examination for early detection of oral cancer. By enhancing awareness and empowering individuals, it is possible to improve oral health outcomes and reduce the burden of oral cancer.

Therefore, the current study aimed to evaluate the effectiveness of a health promotion intervention to improve the knowledge and change the tobacco chewing and self-mouth examination practices among a group of vulnerable youth residing in urban slum areas in district of Colombo, Sri Lanka.

## Methods

The study was a community based quasi experimental study, conducted in the urban slums in the district of Colombo, Sri Lanka in the period of year 2017 to 2018. The study was conducted in 3 phases namely pre intervention assessment, implementation of the intervention and post intervention assessment after 6 months. Study participants were youth participants aged between 15 and 24 years old residing in relevant urban slums. Intervention group (IG) and the control group (CG) was selected from a list of Grama Niladari (GN) divisions in district of Colombo considering the feasibility issues, sociodemographic and baseline characteristics. According to the Rothman and Greenland when the intervention is confined to two study areas random selection is not required as the sole purpose is to ensure the comparability of background variables to minimize the confounding [20]. To minimize contamination GN areas for IG and CG were selected which were situated at a long distance. Urban slums were selected randomly from a list of urban slums within the selected GN divisions. Cluster sampling technique was utilized, and sample size was calculated using a formular [21] with alpha error equals to 0.05 and power equals to 0.90. Sample size for one group was 120 participants. Cluster size was 20 and number of clusters came as 6. One cluster was considered as a one urban slum area.

A pre-tested, validated interviewer-administered questionnaire was used to gather the relevant information. Knowledge was assessed using 10 statements regarding the awareness of oral cancer and oral potentially malignant disorders, clinical features of oral potentially malignant disorders, risk factors and self-mouth examination practices. Practices of tobacco chewing was assessed using betel quid chewing and commercially prepared tobacco and areca nut packet (CPTAP) chewing. A current chewer was defined as a participant who had the chewing lifestyle during past 30 days before the survey [11, 12].

Health Promotion intervention was implemented as a multicomponent package including advocacy, interactive discussions, introduction of Information Education Communication (IEC) materials and community mobilization. Main groups used in community mobilization was the youth societies. Community leaders and youth leaders played a major role. Post intervention assessment was conducted after 6 months following the implementation of the intervention.

Data collection was carried out after obtaining the written informed consent and statistical analysis was done using the SPSS version 21. Post intervention assessment was conducted, using the same study instrument and same data collectors. Socio demographic characteristics between the groups were analysed using frequencies and percentages and tested using X^2^ test, fisher exact test and independent sample Mann Whiteney U test. Effectiveness of the intervention programme evaluated by the difference between the groups, analysing the knowledge differences, the quit rate (proportion of youth who became non-users at end line from among those who were current users in the baseline survey) and fresh up take rate (proportion of youth who reported themselves as non- current users or current users at end line from among those who were never users at the baseline survey) of tobacco chewing. P value < 0.05 was considered as statistically significant. Ethical approval was taken from the Faculty of Medicine, University of Colombo, Sri Lanka.

## Results

A total of 240 youth was included in the sample and all the components of the intervention package were implemented and monitored within the intervention group. Baseline sociodemographic characteristics of the IG and the CG was similar and not statistically different between the group. Socio demographic characteristics of the intervention group and the control group is described in the Table 1.

**Table 1:** Socio demographic profile of the study sample

Variable		Intervention Group N = 120 No (%)	Control Group N = 120 No (%)	P value
Mean Age (Standard deviation)		16 years (1.9 years)	15.6 years (1.4 years)	0.18
Sex	Male	61 (51%)	69 (57%)	0.36
	Female	59 (49%)	51 (43%)	
Ethnicity	Sinhala	71 (59%)	87 (72%)	0.09
	Tamil	31 (26%)	21 (17%)	
	Muslim	18 (15%)	12 (10%)	
Employment status	School student	58 (48.3%)	63 (52.5%)	0.32
	Employed	27 (22.5%)	18 (15%)	
	Unemployed	35 (29.2%)	39 (32.5%)	

Post intervention assessment was done after 6 months. Response rate in the post intervention assessment was 100% and there was no loss to follow up.

Mean Knowledge score for the intervention group was 4.4 (95% CI 4.0-4.7) and for the control group it was 4.6 (95% CI 4.2%-5.0%) at the pre intervention assessment and this difference was not significant, P = 0.264. Mean knowledge score in post intervention assessment for intervention group and control group was 7.2 (95% CI 6.7–7.5) and 4.7 (95% CI 4.4-5.0) respectively. Knowledge score was significantly differed between the groups at post intervention assessment, P = 0.000. Knowledge score was significantly higher among the females in both groups in the pre intervention assessment and post intervention assessment.

Prevalence of current tobacco chewers in the intervention group and the control group was similar (40%) in pre intervention assessment and in post intervention assessment it has reduced up to 26.7% in the intervention group and increased up to 51.7% in the control group. Considering the betel chewing practice and CPTAP chewing practice separately, betel chewing practice has reduced markedly in intervention group compared to the CPTAP chewing practice. Table 2 showed the tobacco chewing practices between the groups. Tables 3 and 4 explained the tobacco chewing practice within the different sexes in intervention group and control group. Tobacco chewing practices are significantly higher among male sex in both pre and post intervention assessments.

**Table 2:** Tobacco Chewing practices among study sample

	Pre-Assessment			Post-Assessment		
	IG N = 120 No (%)	CG N = 120 No (%)	P value	IG N = 120 No (%)	CG N = 120 No (%)	P value
Tobacco chewing practice						
CU	48 (40%)	48 (40%)	0.872	32 (26.7%)	62 (51.7%)	0.001
NCU	27 (22.5%)	24 (20%)		46 (38.3%)	28 (23.3%)	
NU	45 (37.5%)	48 (40%)		42 (35%)	30 (25%)	
Betel chewing practice						
CU	20 (16.7%)	21 (17.5%)	0.983	6 (5%)	27 (22.5%)	0.001
NCU	45 (37.5%)	45 (37.5%)		62 (51.7%)	54 (45%)	
NU	55 (45.8%)	54 (45%)		52 (43.3%)	39 (32.5%)	
Tobacco and areca nut packet chewing practice						
CU	31 (25.8%)	33 (27.5%)	0.871	27 (22.5%)	43 (35.8%)	0.071
NCU	20 (16.7%)	22 (18.3%)		24 (20%)	18 (15%)	
NU	69 (57.5%)	65 (54.2%)		69 (57.5%)	59 (25%)	

CU- Current user, NCU-Non-Current user, NU-Never user

IG-Intervention group, CG-Control Group

**Table 3:** Tobacco chewing practice within the different sexes in the intervention group

	Pre-Assessment			Post-Assessment		
	Male N = 61 No (%)	Female N = 59 No (%)	P value	Male N = 61 No (%)	Female N = 59 No (%)	P value
Tobacco chewing practice						
CU	35 (73%)	13(27%)	0.000	32 (100%)	0	0.000
NCU	16 (59%)	11 (41%)		19 (41%)	27 (59%)	
NU	10 (22%)	35 (78%)		10 (24%)	32 (76%)	
Betel chewing practice						
CU	7 (35%)	13 (65%)	0.000	6 (100%)	0	0.008
NCU	34 (76%)	11 (24%)		35 (57%)	27(43%)	
NU	20 (36%)	35 (64%)		20 (38%)	32(62%)	
Tobacco and areca nut packet chewing practice						
CU	31 (100%)	0	0.000	27 (100%)	0	0.000
NCU	20 (100%)	0		24 (100%)	0	
NU	10 (15%)	59 (85%)		10 (15%)	59 (85%)	

CU- Current user, NCU-Non-Current user, NU-Never user

**Table 4:** Tobacco chewing practice within the different sexes in the control group

	Pre-Assessment			Post-Assessment		
	Male N = 69 No (%)	Female N = 51 No (%)	P value	Male N = 69 No (%)	Female N = 51 No (%)	P value
Tobacco chewing practice						
CU	39 (81%)	9 (19%)	0.000	49 (79%)	13 (21%)	0.000
NCU	19 (79%)	5 (21%)		16 (57%)	12 (43%)	
NU	11 (23%)	37 (77%)		4 (13%)	26 (87%)	
Betel chewing practice						
CU	12 (57%)	9 (43%)	0.000	14 (52%)	13 (48%)	0.000
NCU	43 (96%)	2 (4%)		45 (83%)	9 (17%)	
NU	14 (26%)	40 (74%)		10 (26%)	29 (74%)	
Tobacco and areca nut packet chewing practice						
CU	33 (100%)	0	0.000	43 (100%)	0	0.000
NCU	19 (86%)	3 (14%)		15 (83%)	3 (17%)	
NU	17 (26%)	48 (74%)		11 (19%)	48 (81%)	

CU- Current user, NCU-Non-Current user, NU-Never user

There were 16 tobacco chewers, 14 betel chewers and 4 CPTAP chewers who quit their practice after the intervention in the intervention group. However, there were no quitters in control group at the end line of the intervention. Quit rate of the tobacco chewing practice, betel chewing practice and CPTAP chewing practice among intervention group was 33%, 70%, and 13% respectively and these quit rates were significantly differed from the control group, P = 0.001.

Fresh up take rate of tobacco chewing in the intervention group was 6.7% compared to the 37.5% in the control group, which was significantly differ between the groups, P = 0.001. When the two practices compared separately, fresh up take rate for betel chewing in intervention group was 5.4% compared to the 27.8% in the control group, P = 0.003. There were no new users of CPTAP after the intervention, in the intervention group compared to the 6 new users in the control group, P = 0.015.

Practice of doing self-mouth examination to early identification of any abnormal symptoms inside the oral cavity was significantly higher in the intervention group after the intervention P = 0.001. This practice was significantly higher among females in the post assessment in intervention group, p = 0.000. Table 5 showed the practice of doing self-mouth examination in pre and post intervention assessment.

**Table 5:** Practice of doing self-mouth examination in pre and post intervention assessment in intervention group (IG) and control group (CG)

Pre-Assessment					Post-Assessment				
Practice of doing self-mouth examination				P value	Practice of doing self-mouth examination				P value
Total, No (%)		Male No %	Female No %		Total, No (%)		Male No %	Female No %	
IG	2 (1.7%)	0	2 (100%)	0.243	IG	50 (41.7%)	10 (20%)	40 (80%)	0.000
CG	2 (1.7%)	0	2 (100%)	0.179	CG	2 (1.7%)	0	2 (100%)	0.179
P = 0.689					P = 0.000				

## Discussion

This study determined basically the effectiveness of a health promotion intervention package on knowledge and selected practices related with oral cancer among 15–24-year-old age youth residing in urban slum areas in Colombo district Sri Lanka. Present study identified that the multicomponent intervention package was effective in improving the knowledge related to oral cancer, increasing the self-mouth examination practices, and reducing the prevalence of tobacco chewing practices. Importantly, the study emphasized combination of various components in the intervention package proved to be more successful in achieving positive outcomes [22]. Intervention was implemented according to the pre plan, and monitoring was done regularly, and it ensured the internal validity of the intervention process [23].

These findings are compatible with other studies conducted in globally. A study conducted in India to test the efficacy of a community-based intervention for tobacco prevention among adolescents in two low socio-economic communities, has used peer leaders, community leaders and local non-governmental organizations as stakeholders and the intervention was comprised of displayed posters, audio and video films, lectures, street plays and rally and distribution of information, communication and education materials. The results of the study have suggested that risk of fresh up take of tobacco at the end of the intervention was 6 times higher in the control group [24]. Further another study conducted in Saudi Arabia in 2014, to find out the effectiveness of an intervention to improve the knowledge of oral cancer, identified that the intervention was significantly effective in improving the knowledge among youth. They have used a lecture, and education brochure and question answer session as the intervention package [25]. Moreover, a quasi-experimental study conducted in 2013 among adults in urban slums in India with a health education intervention related to oral cancer has found that awareness was increased significantly after the health education intervention [26] and a school based cluster randomized intervention study conducted among students in school grade 6–10 in Karachchi, India has determined that knowledge and perceptions about smokeless tobacco used has increased significantly in intervention group [27].

However, some studies identified that even though, knowledge improvement after the intervention, no changes in tobacco using practices [28]. Behaviour change is not a straightforward process, all the literature also supports that multiple efforts should be used to change a behaviour. Present study also used few strategies and multiple activities. Health promotional intervention was developed based on the results of a qualitative study conducted within the urban slum areas in the district of Colombo, Sri Lanka but not in the GN divisions where this present intervention study was conducted. Therefore, intervention was more effective and sustainable.

The present study used interactive discussions with slide shows to give the facts regarding the risk factors and clinical presentation of OPMD and oral cancer. Verbal as well as pictorial messages used in interactive discussions to strengthen the skills of the participants. As well as intervention used many other techniques such as advocacy, IEC materials, community mobilization to reinforce the knowledge and skills. Multiple efforts resulted in significant effectiveness in the present intervention. The present study was conducted among a youth group, where they may not have gone to an addictive phase of the practices could be another important reason for improvements in quit rates.

A study done among adolescents in low-income settlements in India has found that after implementing a community-based intervention, a significant difference in current use of tobacco between the study groups (p = 0.048), with the intervention group showing a reduction in use, compared with an increase in use among the control group. further it reported significantly lower fresh uptake (0.3%) of tobacco in intervention group compared with the control group (1.7%). No significant change was found for quit rate (p = 0.282) between the two groups [15].

One of the limitations in the present study was that intervention group and the control group were selected intentionally and that can introduce selection bias, but within the group slums were selected using random sampling to minimise the bias. Present study included the urban slums in only one district in Sri Lanka, so the picture can be different from other districts. But according to the national census data, this district is highly populated, most of the urban slums are situated in this district, and this is a multi-racial city, where participants represented by all ethnic groups and different socio-cultural strata. Present study has assessed only the short-term effects after 6 months. Long term effects have not assessed. A systematic review conducted in year 2021 has identified that the individual or community interventions to improve the knowledge of oral cancer were generally effective among general public as well as high risk groups, but long-term benefits were still understudied [3]. Further research needed to assess the long-term effects as well as the effectiveness in other parts of the country.

## Conclusion

Multicomponent health promotion intervention (Advocacy, Interactive discussions, IEC materials and Community mobilization) was significantly effective to improve the knowledge and changed the tobacco chewing and self-mouth examination practices among a vulnerable group of youth in Sri Lanka.

## Electronic supplementary material

Below is the link to the electronic supplementary material.

Supplementary Material 1

## Data Availability

Available as a supplementary material.
